# Inspecting the potential physiological and biomedical value of 44 conserved uncharacterised proteins of *Streptococcus pneumoniae*

**DOI:** 10.1186/1471-2164-15-652

**Published:** 2014-08-05

**Authors:** Antonio J Martín-Galiano, José Yuste, María I Cercenado, Adela G de la Campa

**Affiliations:** Centro Nacional de Microbiología and CIBERES (CIBER de Enfermedades Respiratorias), Instituto de Salud Carlos III, Majadahonda, Madrid, Spain; Presidencia, Consejo Superior de Investigaciones Científicas, Madrid, Spain

**Keywords:** Antibiotic target, Bacterial pathogenesis, Hypothetical protein, Post-genomics, Protein function, Protein space, Proteomics, Virulence factors

## Abstract

**Background:**

The major Gram-positive coccoid pathogens cause similar invasive diseases and show high rates of antimicrobial resistance. Uncharacterised proteins shared by these organisms may be involved in virulence or be targets for antimicrobial therapy.

**Results:**

Forty four uncharacterised proteins from *Streptococcus pneumoniae* with homologues in *Enterococcus faecalis* and/or *Staphylococcus aureus* were selected for analysis. These proteins showed differences in terms of sequence conservation and number of interacting partners. Twenty eight of these proteins were monodomain proteins and 16 were modular, involving domain combinations and, in many cases, predicted unstructured regions. The genes coding for four of these 44 proteins were essential. Genomic and structural studies showed one of the four essential genes to code for a promising antibacterial target. The strongest impact of gene removal was on monodomain proteins showing high sequence conservation and/or interactions with many other proteins. Eleven out of 40 knockouts (one for each gene) showed growth delay and 10 knockouts presented a chaining phenotype. Five of these chaining mutants showed a lack of putative DNA-binding proteins. This suggest this phenotype results from a loss of overall transcription regulation. Five knockouts showed defective autolysis in response to penicillin and vancomycin, and attenuated virulence in an animal model of sepsis.

**Conclusions:**

Uncharacterised proteins make up a reservoir of polypeptides of different physiological importance and biomedical potential. A promising antibacterial target was identified. Five of the 44 examined proteins seemed to be virulence factors.

**Electronic supplementary material:**

The online version of this article (doi:10.1186/1471-2164-15-652) contains supplementary material, which is available to authorized users.

## Background

The infectious diseases caused by Gram-positive cocci are a major cause of morbidity and mortality worldwide. The extensive use of antibacterial agents has promoted the selection and dissemination of resistant clones of these cocci in hospital and community environments. Among the most worrying are vancomycin-resistant enterococci (*Enterococcus faecalis* and *Enterococcus faecium*), methicillin-resistant *Staphylococcus aureus* and penicillin-nonsusceptible *Streptococcus pneumoniae* [1]. Treatments must now frequently be extended, and therapeutic failure is on the increase. This is not helped by the small number of targets sought out by the antibiotics used in the clinical setting; indeed, our present antibiotic arsenal focuses on just some 25 bacterial proteins (the richest pool of possible targets). Further, only half a dozen new antibacterial agents have reached the market over the last 10 years, and resistance to these was promptly detected in clinical practice [2]. Moreover, these new drugs are associated with undesirable side effects [3, 4] and may suffer inactivation in some parts of the body [5]. The need to discover proteins essential to pathogens that can act as new therapeutic targets is therefore clear.

The roles of many of the proteins apparently involved in the pathobiology of Gram-positive cocci are poorly understood. This is particularly true with respect to the transition from commensal to pathogenic status. Different bacterial pathogens appear to make use of similar strategies to infect their hosts; this is particularly notable among the pathogens that cause pneumonia, sepsis, endocarditis and meningitis [6]. In *S. pneumoniae* and *Haemophilus influenzae*, proteins involved in metabolic pathways leading to coccal chain length reduction to just one or two cells have been related to virulence via the impairment of complement fixation and subsequent opsophagocytosis [7]. A number of pathogens also rely on the autolysis – sometimes non-fatal – of some of their population. This releases highly inflammatory fragments of cell wall and cytoplasmic virulence factors into host tissues, and frees other virulence factors, facilitating invasion by the population as a whole [8–10].

While the molecular basis of these common invasion strategies remains largely unknown, it likely involves the complex interplay of different proteins. Its examination via high-throughput experiments (HTEs) and systems biology techniques is therefore highly desirable. Microarrays are now being used to reveal changes in global transcription under different conditions, signature-tagged mutagenesis (STM) is being used to determine the genes essential under different infective scenarios [11], and “antigenome” techniques [12] are being used to determine the bacterial immunogenic polypeptides recognized by antisera from patients or carrier individuals. Many of the genes shown by these techniques to be involved in pathogenesis encode “hypothetical proteins” (HPs), i.e., those for which no exact function can be inferred. The term ‘HP’ covers the potential polypeptides associated with: 1) open reading frames (ORFs) that code for no protein at all, typically those smaller than 80 codons [13], 2) truncated and degenerated pseudogenes, 3) species- or strain-specific genes (ORFans), 4) remote superfamily homologues, and 5) genes present in many organisms [14, 15]. The wide taxonomic distribution of this fifth type of HP (commonly known as conserved HPs [cHPs]) suggests these proteins could be of great importance to cells. cHPs are a heterogeneous collection of proteins that have proven very difficult to work with in the laboratory, or they have very complex domain combinations that hinder any prediction of functionality. They often contain domains of unknown function (DUFs), classified by the Pfam protein domain resource as domains lacking sufficiently documented activities [16]. Pfam provides a curated library of profile hidden Markov models for 13,672 conserved domain families for which the relative abundance of DUFs increases with every new version (currently n = 3526; ~26% of the total number of models) [17].

Genes poorly characterized, or not characterized at all, account for 28% of the pneumococcal core genome [18]. Many of these have been shown essential for survival *in vitro* [19, 20], in nasal colonization [21], and during the infection of the ear [21], lung [22] and cerebrospinal fluid [23]. However, their contribution to bacterial physiology has not been further analysed, hindering advances in our understanding of how they may be involved in bacterial virulence [24]. In the post-genomic age, orchestrated bioinformatic and biochemical initiatives are required to remedy this lack of knowledge [25]. Such a characterization of the HPs – and especially of the cHPs – encoded would be of enormous value [26]: it would increase the catalogue of protein functions potentially transferable to homologues in other bacteria [15], help identify new virulence factors, and aid in the identification of new antimicrobial targets for medium-spectrum therapy [27].

The present work examines the potential physiological and biomedical importance of 44 selected cHPs from *S. pneumoniae* with homologues in *E. faecalis* and/or *S. aureus*. Different cHPs were found to have different domain architecture and to be differently involved in bacterial growth and morphology. Five cHPs were found to be virulence factors, and one was recognized as a promising antibacterial target.

## Results and discussion

### Selection of conserved hypothetical proteins

**Figure 1:**
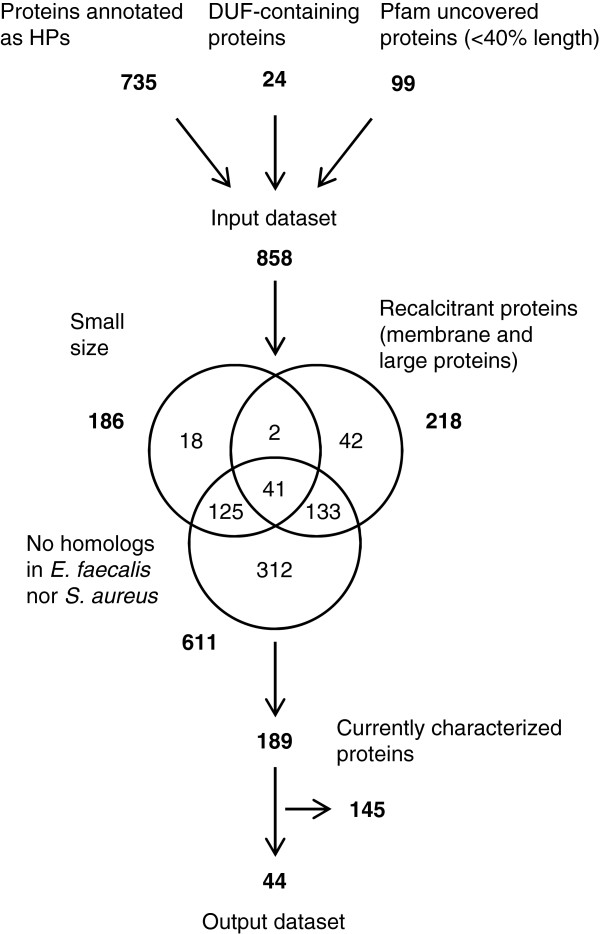
**Protein selection procedure.** The numbers of candidates rejected and accepted during the selection procedure are indicated. Numbers in Venn diagrams indicate proteins rejected by one or more of the corresponding criteria.

*S. pneumoniae* is a major pathogen suitable as a model system for biomedical studies [28]. In order to select cHPs of *S. pneumoniae* R6 that were truly uncharacterised and that were chemically amenable to experimental analysis, 858 potential cHPs were initially selected (Figure 1). These comprised proteins already annotated as HPs, as well as those containing DUF domains or only partially covered (<40% length) by Pfam domains. HPs with a narrow taxonomic distribution, without homologues in *E. faecalis* and *S. aureus*, or of small size (gene-finding algorithms tend to detect false positives in short-length ORFs [13], and experimental information exists for only 30% of proteins with <100 residues [29]), were then rejected. This rejection by size involved all those potential HPs of <80 residues. Those between 80 and 120 residues were not rejected if they met one of the following conditions: (a) mean identity to streptococcal homologues of at least 60%, (b) at least one HTE hit (see below), or (c) the possession of two or more cysteine residues (which can form disulphide bridges) in the amino acidic sequence. Finally, those HPs showing evidence of being difficult to handle experimentally were also rejected, i.e., large (>800 residues) and membrane-embedded proteins. However, those membrane proteins with ≤2 transmembrane helices plus a contiguous span of ≥100 non-membrane residues were contemplated in the analysis. These exclusions led to a list of 189 HPs. Using the BLAST tool, their current annotation status was manually checked against the Uniprot database [30], and their domain architecture checked using the Pfam domain organization database. Certainly, the available *S. pneumoniae* R6 annotation which was published 12 years ago is now largely obsolete [31], and although many of the HPs examined had consistent functional annotations, 44 (~2% of the pneumococcal proteome) (Additional file 1: Table S1) remained uncharacterised, annotated by vague descriptors, or simply associated functionally to promiscuous superfamilies (a common cause of miss-annotation [32]). For example, Spr0705 belongs to the ASCH superfamily and Spr1424 to the P-loop ATPases superfamily. These superfamilies have different roles in RNA binding/metabolism [33] and macromolecule remodelling [34] respectively, which prevents direct functional annotation. The 44 uncharacterised proteins, several of which are apparently nucleic acid (either DNA or RNA) binding proteins (common among small DUF proteins [35]) (Additional file 2: Table S2), were selected for further analysis.

### Classification of the 44 selected cHPs into 4 classes based on domain architecture, sequence conservation and interactivity

**Figure 2:**
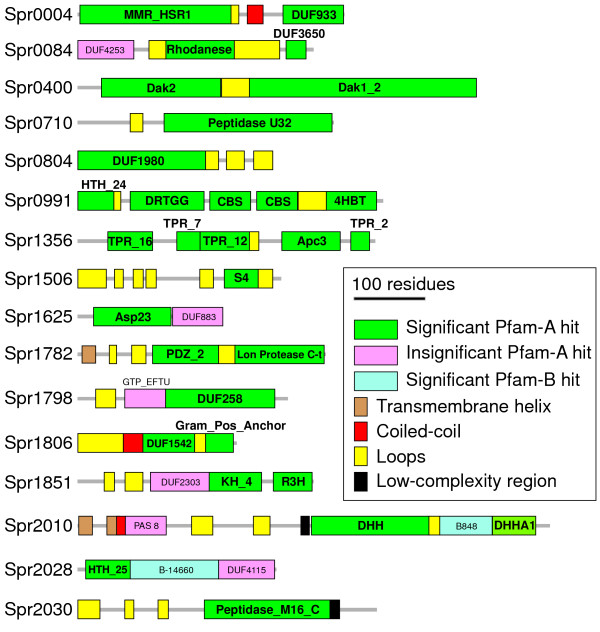
**Domain and motif architecture of modular proteins.** Sequence domain and motifs were successively mapped on the sequence using the prevalence system established by the top-down order indicated in the inset. Elements were only considered if they did not overlap by more than 10 residues with a previously established element. The Pfam domain description, location and E-values are provided in Additional file 7: Table S4.

The mapping of the Pfam domains in the 44 proteins revealed two architectural classes. The first class, DUF proteins, was composed of 28 rather small (188 ± 84 residues, average ± SD) monodomain cHPs; a single Pfam profile occupied most of their entire length (82.8% ± 15.4). The second class, modular proteins, involved 16 (presumably) multidomain proteins with either ≥2 Pfam domains or 1 Pfam domain plus additional unclassified sections long enough to be a domain (≥70 residues) (Figure 2) (Additional file 3). Such proteins typically contain promiscuous domains of known general activity (*e.g*., protease or cell-wall anchoring functions) that tend to combine with other domains to endow novel functionalities [36]. Modular proteins are, on average, twice as long as DUF proteins (377 ± 111 residues), and may have complex architecture (such as the pentadomain Spr0991 protein) and even contain DUF domains. The Pfam profiles only covered 55.9 ± 19.3% of the length of the modular proteins when the gathering thresholds recommended by the Pfam administrators were taken into account (significant Pfam-A hits in Figure 2). The nature of the remaining unclassified regions was subjected to: 1) the detection of additional Pfam domains with low E-values (<0.01), even though they did not satisfy their respective gathering thresholds (insignificant Pfam-A hits) (these may be considered remote homologues of the given families); 2) searching for other regions covered by the Pfam-B database (significant Pfam-B hits), a non-curated additional Pfam database containing domain families with a typically narrow taxonomic distribution; and 3) the detection of any segment of any remaining section predicted to be unstructured, a coiled-coil, or as having low-complexity residue composition. A high concentration of these kinds of element in a given protein section is suggestive of it having a role that requires there be fewer structural constraints, e.g., when acting as a dimerization zone or flexible stalk.

**Figure 3:**
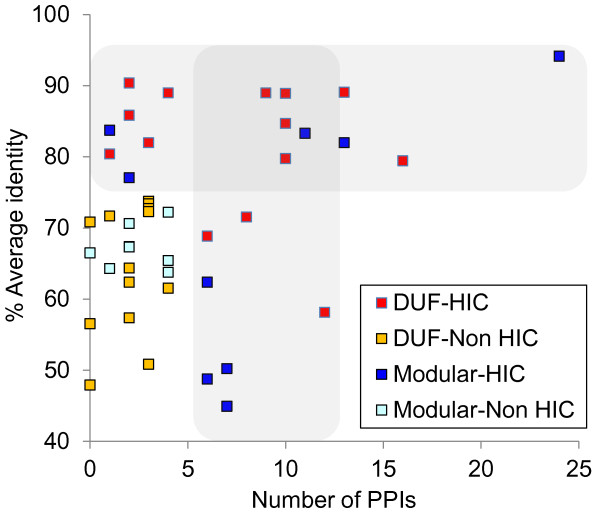
**Protein sequence conservation in streptococci and estimated number of protein-protein interactions (PPIs).** The graph areas corresponding to HIC proteins are shadowed.

High sequence conservation [37] and interaction with many other protein partners [38] provide indirect proof of biological importance. The 44 selected cHPs differed in terms of sequence identity with homologues in other streptococci and the number of predicted interacting partners (Figure 3, Additional file 1: Table S1). Twenty three cHPs showed ≥75% identity to their streptococcal homologues and/or ≥6 protein-protein interactions (PPIs) (i.e., they were highly interactive and/or sequence conserved proteins [HIC]; located in the shadowed areas of Figure 3). These HIC proteins would be expected to play basic roles in the physiology of Gram-positive cocci. A four-class classification of cHPs – DUF-HIC, DUF-Non HIC, modular-HIC and modular-Non HIC – is hereafter used to describe these cHPs.

### cHPs and high-throughput experiments: appearance in the literature

Many bacterial pathogens occupy a number of niches in humans. HTEs can detect genes important for the successful colonization of new environments. The results of microarray experiments on *S. pneumoniae* performed under 27 environmental conditions, of STMs involving ear, lung, nasal and meningial infection, and of one antigenome experiment were examined (Additional file 4: Table S3). Thirty five out of the 44 cHPs studied appeared in 1–6 conditions (Additional file 5: Figure S1). A normalized HTE score was then derived ranging from 0.25 to 5 (see Methods). For Non-HIC proteins, the HTE score was, on average, twice that of the HIC proteins (1.58 vs 0.77). The association between high HTE scores and the Non-HIC class suggests these proteins play accessory functions adaptable to specific conditions rather than constant housekeeping activities.

### Gene essentiality and protein druggability: the *spr0479*gene encodes a promising antibacterial target

**Figure 4:**
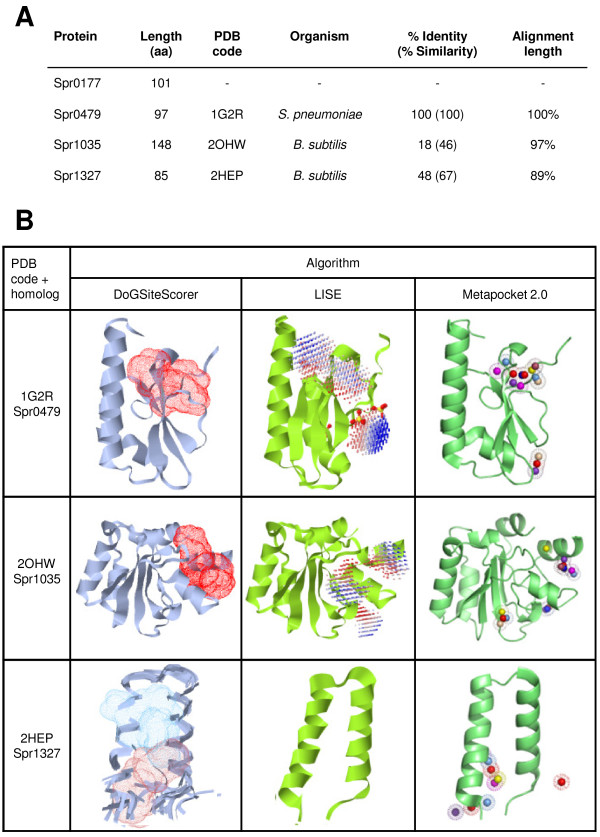
**Structural analyses of cHPs encoded by potential essential genes.** **(A)** Data for the closest homologue with a structure in the Protein Data Bank (PDB). **(B)** Pockets predicted by two servers and one meta-server (nine algorithms in total).

To assess the biological importance of the selected cHPs, the encoding genes were substituted by a chloramphenicol resistance cassette by double recombination. Forty knockout mutants were obtained (transformation rate >10^4^ CFU ml^−1^), but no viable knockouts were obtained for *spr0177*, *spr0479*, *spr1035* and *spr1327* even after three attempts (transformation rate <10^2^ CFU ml^−1^); these genes were therefore classified as potentially essential. These genes may encode cHPs that could be used as targets in antimicrobial therapy. However, an ideal target must also be druggable, i.e., it must be able to bind ligands that modulate the protein’s function, and this must eventually lead to the bacterium’s death, or at least the prevention of its growth. The existence and availability of a high-quality structure for at least one homologue, a condition fulfilled by 3 of the 4 potentially essential cHPs (Figure 4A), is an indispensable prerequisite for the detection of potential drug-binding cavities. In order to cover the range of binding-pocket structures, and the different chemical properties of their natural ligands, a consensus of nine independent strategies was used: the seven algorithms of Metapocket 2.0 [39], and the DoGSiteScorer [40] and LISE [41] algorithms (Figure 4B).

Despite the fact that three well-defined pockets were found in *Bacillus subtilis* YueI protein, the homologue structurally resolved closest to Spr1035, the lack of identity between these proteins (18%) suggests drugs against this protein family would only have a narrow spectrum of activity. The next essential cHP studied, Spr1327 showed strong identity (48%) to the putative stress protein YnzC from *B. subtilis* [42]. Although DoGSiteScorer divided the large interhelical cavity into two parts, LISE and Metapocket failed to find any consistent pocket in this structure; this protein was therefore deemed non-drugable.

In contrast, there is evidence that suggests Spr0479 may be a promising target for rational drug design. The structure of Spr0479 is known at high resolution (1.35 Å) [43], and provides an excellent dataset of atomic coordinates. A cleft has been found by all the cavity-detection algorithms used. Spr0479 is predicted to interact with proteins involved in translation (such as initiation factor IF-2), one of the processes most commonly targeted by antibacterial agents. The Spr0479 sequence shares 40-51% identity (64-70% similarity) with orthologues from Gram-positive pathogens highly recalcitrant to antibiotic therapy, such as *Clostridium difficile*, *E. faecium* and *S. aureus*. In addition, the Pfam family of Spr0479 has no members in the *Homo sapiens* proteome. To ascertain the essentiality of the *spr0479* gene, an ectopic additional gene copy under a Zn-inducible promoter was introduced into a disposable chromosomal site (see Methods for details). The native *spr0479* gene copy could not be removed (<10^3^ transformants ml^−1^) unless 10 μM ZnCl_2_ was added to the medium (3.8 ± 0.8 × 10^5^ transformants ml^−1^, n = 2). These results indicate that the second gene copy rescued cell viability in a Zn-inducible manner, and explicitly confirms the essentiality of *spr0479*. Future investigations on chemical ligands binding to Spr0479 may allow the design of new antibacterial agents that target this essential protein.

### Some viable knockouts grow more slowly and/or show a chaining phenotype

The growth rate and cell morphologies of knockout mutants for non-essential genes were then examined. Two of them, Δ*spr0391* and Δ*spr0399*, were able to grow in the semi-synthetic pneumococcal-specific AGCH-SYE medium, but grew deficiently in the more universal THYE medium (OD_620_ < 0.2 after 4 h growth under the present experimental conditions [see Methods]). These knockouts were therefore classified as “medium-dependent”. A correlation was seen between the severity of the mutant phenotype and the protein class involved. Genes coding for DUF-HIC proteins were over-represented in the lethal or medium-dependent knockouts obtained since five of the six genes involved belong to this class (p = 0.009; Fisher’s exact test). The reduction in biological fitness observed in HIC knockouts suggests that these proteins play fundamental roles. Similarly, proteins central to the interactome network of *Saccharomyces cerevisiae* are often essential for its viability [38]. Further, PPIs have been used to detect putative antimicrobial targets in *Pseudomonas aeruginosa* [44]. In contrast, non-HIC proteins may be more physiologically isolated, *i.e*., adapted to more specific roles under particular conditions (as suggested by their higher HTE scores). Thus, roles may be inferred for HIC and Non-HIC proteins as antimicrobial targets and virulence factors respectively.

**Figure 5:**
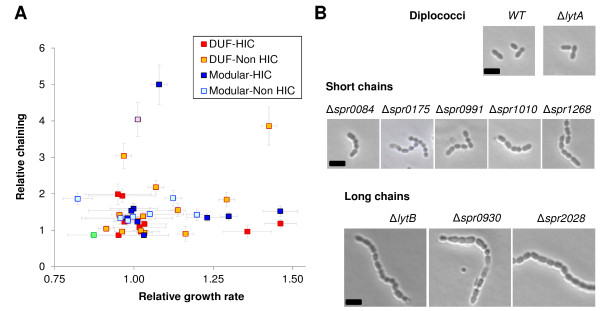
**Cell growth and chaining morphology of viable knockouts.** **(A)** Growth rate versus length expressed in number of diplococcal units. Both values are relative to the original R6 strain. The controls Δ*lytA* (green square) and Δ*lytB* (rose square) are also shown. **(B)** Photograph panel of representative knockout specimens that tend to grow as short or long chains. Only deletion mutants without significant growth delays are shown. Bar represents 2 μm.

Knockouts for 11 of the genes had duplication times 10-46% longer than that of the wild type (Figure 5A). One of the slowest knockouts, Δ*spr0004*, was reported non-viable in one study [20] but viable in another [19], underscoring the importance of the experimental setup when defining essentiality.

All the knockouts viable in THYE medium (n = 38) were visualized by optical microscopy and the average number of coccoid units per chain calculated. While the wild type grew mainly in a diplococcal fashion (about 80%; mean chain length = 1.21 diplococcal units [60 specimens examined]; Figure 5B, top panel), 10 of the 38 knockouts showed longer morphologies (mean chain length >1.94 diplococcal units; p<10^−4^ [two-tailed unpaired Student t test]). Of these, seven knockouts formed short chains (average <3 diplococcal units; Figure 5B middle panel) and three formed long chains (average >3 diplococcal units; Figure 5B bottom panel). The severe separation defect of these last three mutants is similar to that seen for Δ*lytB*, which is deficient in a protein involved in the separation of daughter cells [45].

The genes deleted in five of the 10 chaining knockouts presumably encode DNA-binding proteins (Additional file 2: Table S2). The lack of these proteins might cause epistatic effects leading to chaining via the loss of the regulatory transcriptional equilibrium that maintains diplococcal morphology. Similar results have been reported by Dahlia and Weisser, who found an abundance of genes coding for either regulators or enzymes in random knockouts with defective diplococcal separation [7]. Chaining would therefore appear to be a meta-phenotype reachable via several direct (*e.g*., lack of enzymes related to cell wall metabolism) or indirect (*e.g*., lack of regulators) alterations.

### Some chaining knockouts show defective autolysis

**Figure 6:**
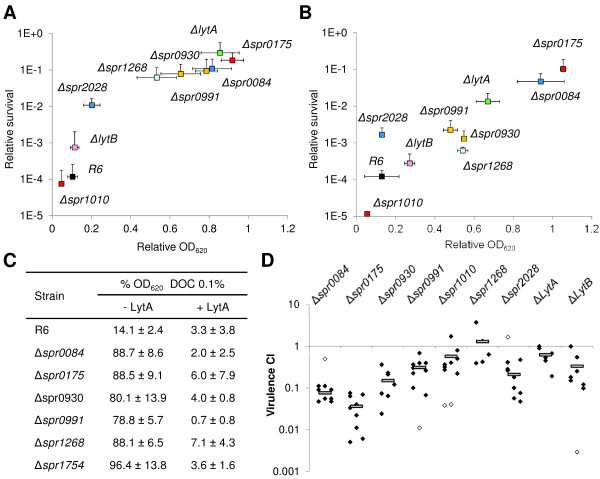
**Autolytic and virulence capacities of knockouts.** Vancomycin **(A)**, penicillin **(B)** and DOC **(C)** treatments. DOC assays were performed with (for 5 min) and without (for 30 min) the addition of exogenous LytA. Means ± SD for experiments performed in triplicate for **(A)** and **(B)**, and in duplicate for **(C)**, are shown. Colours for protein classes are as described in the legend to Figure 5. **(D)** Virulence capacity of knockouts. Grey bars represent the average CI with respect to the D39 strain in an *in vivo* sepsis model. Diamonds represent the CI value of a single mouse. Values 10-fold higher or lower than the average (white diamonds) were not considered in calculations of the average CI (up to a maximum of 2 mice per knockout).

Since modifications to the cell wall typically cause a chaining phenotype and reduce susceptibility to antibiotics targeting enzymes involved in peptidoglycan biosynthesis [46], cultures of chaining knockouts showing normal growth (n = 7) were challenged with either vancomycin or penicillin. Both these antibiotics reduced the optical density (OD_620_) of a wild type culture by 10-fold, and cell viability by 4 orders of magnitude. In the presence of vancomycin, five of the knockouts showed an OD_620_ reduced by 50% after 2 h, and a survival rate reduced by <2 orders of magnitude, in a similar fashion to the Δ*lytA* knockout (defective for autolysin) (Figure 6A). These results support that idea the vancomycin tolerance phenotype involves several genes [47]. In the presence of penicillin, two of these five knockouts (Δ*spr0084* and Δ*spr0175*) showed no reduction in OD_620_ and survival was only diminished by one order of magnitude (strongly defective autolysis); the remaining three (Δ*spr1268*, Δspr*0930* and Δspr*0991*) showed ~2-fold reductions in OD_620_ and reduction of three orders of magnitude in survival (partially defective autolysis) (Figure 6B). This dual vancomycin and penicillin tolerance has also been observed in certain clinical isolates [46]. Only the Δspr*0991* knockout appeared to have lost a putative DNA-binding protein; the others likely lack enzymes directly affecting the composition, shape or thickness of the cell wall. Cell wall status was therefore further assessed by treating these knockouts with 0.1% deoxycholate (DOC), a bile salt that induces LytA-mediated lysis. All five knockouts were DOC-resistant, suggesting the presence of an altered cell wall, which may require more LytA protein to lyse the cell than that natively produced. To check this, cultures were pre-treated with exogenous pneumococcal LytA prior to DOC-treatment. In all cases, the cells underwent autolysis within 5 min of adding the DOC (Figure 6C), suggesting that the modified cell walls can still bind LytA and remain valid chemical substrates for this enzyme, although more is needed for lysis to occur. These findings also support the notion that defective autolysis is another meta-phenotype, like chaining, that results from the alteration of one or more several possible pathways.

### Some knockouts showed attenuated virulence

Since the selected knockouts had different combinations of chain length and lysis defects, their relative effect on infectivity was examined. Equivalent knockouts were constructed in the highly virulent D39 strain (IU1680), the pathogenic parental of R6. The ability of these mutants to cause sepsis was evaluated. Values significantly below 1 indicate that deletion causes the attenuation of pathogenesis. The control knockouts for defective autolysis and cell separation, Δ*lytA* and Δ*lytB* respectively, were slightly attenuated in their ability to compete with the wild type (Figure 6D), confirming that the respective proteins contribute to pneumococcal pathogenesis. Although LytB is involved in preventing phagocytosis, in particular when combined with LytC [48], there is some controversy regarding the contribution of LytA towards pneumococcal pathogenesis in sepsis models. Some authors suggest it has no effect [49], while others report it to reduce bacterial titres by four logarithmic units [50] – differences that might, however, be explained by experimental procedure. Nevertheless, the slight reduction in virulence observed in the Δ*lytA* and Δ*lytB* control knockouts under the present conditions is optimal for quantifying additional infectivity loss when defective autolysis and chaining are combined in a single strain. Strong attenuation (CI < 0.2) was observed for Δ*spr0084* and Δ*spr0175* (which combine both short chains and strongly defective autolysis), and for Δ*spr0930* (long chains, partially defective autolysis), suggesting that these genes play important roles in pathogenesis. Moderate attenuation (0.2 < CI < 0.4) was seen in Δ*spr0991* (short chains, partially defective autolysis) and Δ*spr2028* (long chains, no defective autolysis). Only slight attenuation (CI ~ 0.6) was observed for Δ*spr*1010 (short chains, no defective autolysis) and no attenuation for Δ*spr1268* (short chains, partially defective autolysis). The results for Δ*spr1268* underscore the idea that while chaining and autolysis are important facets of virulence, they function in concert with other factors that might counteract them. Nevertheless, chaining and defective autolysis do appear to have an apparent synergistic effect on sepsis. It is worth remembering that these knockouts had generation times similar to the wild type; their low CIs can therefore can be attributed to a genuine reduction in virulence rather than a global loss of biological fitness.

Our lack of precise knowledge regarding the contribution of these proteins to cell wall metabolism prevents any straightforward interpretation of the present results. However, the rhodanase-like domain detected in Spr0084, a domain present in a superfamily of enzymes involved in sulphur reactions [51], suggests that this protein might be involved in sulphur metabolism. In addition, Spr0930 shares remote homology with lysozymes, although its exact biochemical activities and cellular role remain to be elucidated. Spr0930 is a putative outer protein, given that it has signal peptide and immunogenic properties [52].

## Conclusions

This paper reports an attempt to characterize the genes coding for cHPs in Gram-positive cocci using *S. pneumoniae* as a model organism. These proteins were organized into two architectural groups, *i.e*., monodomain DUFs and modular, and two potential levels of importance in terms of sequence conservation and interaction, *i.e*., HIC and Non-HIC proteins. Deletion of HIC-protein-encoding genes suggests their products often play central physiological roles. In contrast, Non-HIC proteins would seem to be more related to adaptation to infective conditions.

Spr0479 is a cHP that might have potential as a novel target for antibiotherapy. It is essential for bacterial growth and is predicted to interact with protein partners involved in translation. Its crystal structure shows a cleft with drugability potential, and its high sequence conservation across bacterial pathogens makes it attractive as a therapeutic target. In addition, five proteins – Spr0084, Spr0175, Spr0930, Spr0991 and Spr2028 – that might participate in cell wall metabolism were found involved in pathogenesis. Their respective knockouts lost classic diplococcal morphology and they could not effectively undergo autolysis, two properties required for full virulence to be realised. Finally, virulence factors Spr0084 and Spr0930 are two apparent cell wall enzymes with a small number of interacting partners and high HTE-scores; these proteins may act in concert in several organisms to provide a physiological background in which host invasion becomes more efficient.

## Methods

### Ethics statement

The animal experiments performed in this work were approved by the Animal Care and Use Committee of the *Instituto de Salud Carlos III* (CBA PA 52_2011-v2).

### Sequence collection and bioinformatic methods

Genomic sequences were downloaded from the NCBI FTP site (http://www.ncbi.nlm.nih.gov/Ftp/). The *S. pneumoniae* R6 sequence [31] was used as a reference. Homologues were searched for by BLAST [53] within the Uniprot database [30]. Pfam domains were located using the search tool available on the Pfam web server (http://pfam.xfam.org/search). A protein sequence was considered annotated if ≥5% BLAST hits had the same assigned functions or only trivial semantic variations (*e.g*., “DNA replication protein dnaD” and “Chromosome replication initiation protein dnaD”). This threshold was chosen after careful inspection of updated Pfam domain descriptions and the literature in Pubmed. Only the top 1000 hits with E-values of ≤10^−10^ and ≥30% identity were analysed. An alignment of >60% of the total protein length was demanded to avoid spurious functional assignation caused by mobile domains, which can be found in different domain architectures. Monodomain proteins with an apparent function were considered annotated unless manual inspection revealed the domain to be either pending true annotation or associated with a large variety of activities that prevented the inference of a precise function. Transmembrane helices were predicted using Phobius [54], unstructured regions with the DisEMBL algorithm, [55] and low-complexity sequences with the SEG algorithm [56].

Average streptococcal identities were calculated using the closest homologue (best BLAST mutual hit) from 12 streptococcal species (44 strains with complete genome sequences) (Additional file 6). Sequence identity was multiplied by the length of the alignment relative to the total protein length (≤1), which penalizes non-aligned regions. The number of PPIs was taken from the STRING database, setting a score threshold of 0.7 (confident interaction level) [57]. Homologues with available structures were downloaded from the PDB FTP site. To derive the HTE-score, all hits in published works on HTE (≥20 genes) (Additional file 4: Table S3) were taken into account. This count was further normalized by awarding 1 point to microarray-detected upregulated genes, STM and antigenome hits, and 0.5 points to genes downregulated in microarray experiments. Points for upregulated and downregulated genes in microarray experiments were reduced by half (0.5 and 0.25 respectively) if the total number of responsive genes was >300.

### Knockout construction

To construct deletion mutants, genes were replaced by the *cat* (chloramphenicol acetyl transferase) cassette containing the promoter, coding sequence and terminator [58] in the same orientation as the gene removed. To minimize polar effects on the transcription of downstream ORFs, the cassette did not include the transcriptional terminator if the gene was located in the first or intermediate positions of the predicted operons. Moreover, oligonucleotides were designed so as not to remove the coding regions, ribosome-binding sites or the terminators of adjacent genes. Price algorithm operon predictions [59] were downloaded from http://www.microbesonline.org. Terminators were predicted by TransTermHP [60]. Upstream and downstream flanking regions about 500 bp longer than the length of the gene to be deleted were amplified by PCR and cut with either *BamHI*, *NheI* or *XhoI*, and *NotI* respectively. These amplicons were ligated to the *cat* cassette cleaved with the same enzymes. The ligation product was re-amplified using internal oligonucleotides priming 500 bp from the upstream and downstream ends. This rendered a fragment twice the length of the gene plus the length of the *cat* cassette. *S. pneumoniae* was transformed as previously described [61]. Cassette insertion was verified in viable knockouts by PCR using oligonucleotides priming the internal sequence of the cassette and flanking regions (see the oligonucleotide list in Additional file 7).

To transform the D39 (IU1680) strain, 10 × stock cultures were obtained by growing in AGCH medium supplemented with 0.3% sucrose and 0.2% yeast extract (AGCH-SYE) up to OD_620_ = 0.3. They were then chilled in an ice-water bath for 10 min, centrifuged at 3000 × g × 5 min, resuspended in a 1:10 volume with 20% glycerol, and stored at −80°C until use. Stock cells were gently thawed on ice and resuspended as a 10-fold dilution in pre-warmed AGCH-SYE containing 0.1 mM CaCl_2_, 0.2% BSA and 25 μg ml^−1^ competence-stimulating peptide. Cells were incubated for 10 min at 37°C and then 100 ng ml^−1^ of a PCR product containing the *cat* cassette plus the flanking zones of the gene to remove were added, followed by 40 min incubation at 30°C and then 70 min at 37°C. Pre-induction with 0.5 μg ml^−1^ chloramphenicol was then allowed for 20 min at 37°C. Cultures were plated onto AGCH-SYE containing 1% agar and 2.5 μg ml^−1^ chloramphenicol, and incubated 16 h at 37°C in a 5% CO_2_ atmosphere. The insertion of the *cat* cassette was verified as in the R6 knockouts.

To confirm the essentiality of the *spr0479* gene, an ectopic copy was introduced into the *spr1806* locus. For this, a synthetic DNA molecule was designed, and synthesized and cloned into pET29 by GenScript Ltd., rendering plasmid pZ0479. The construction contained (in the 5’ to 3’ direction) the following elements: an *EcoRI* target, the Zn-inducible promoter CczcD [62], the AGGAGAG consensus ribosome-binding site, a *SacI* target, the *spr0479* full coding region, a *SalI* target, the transcription terminator from the *atp* operon [63], and a *HindIII* target. This construction was fused to a kanamycin resistance cassette yielding plasmid pZK0479. For this, the kanamycin resistance cassette from pR410 [64] was amplified by PCR, digested with *BamHI* and *EcoRI* (targets included in the oligonucleotide sequences), and ligated to pZ0479 digested with the same enzymes. The whole insert was amplified, cleaved with *BamHI* and *XbaI* and ligated to regions flanking the disposable *spr1806* gene in a three-partner ligation reaction. The construction was introduced into *S. pneumoniae* R6 by genetic transformation. Transformants were selected with 250 μg ml^−1^ kanamycin.

### Microbiological analyses of deletion mutants

To quantify the growth rate *in vitro*, glycerol stocks of cultures grown in AGCH-SYE were inoculated into Todd-Hewitt medium + 0.5% yeast extract (THYE). When the cultures reached OD_620_ = 0.15 they were diluted 1/20 in the same medium and growth followed for 4 h at 20 min intervals. The growth rate was calculated as the slope of the growth curve over the exponential range of OD_620_ = 0.05 to 0.5. For microscopy, cells were grown in AGCH-SYE to OD_620_ = 0.3 and then fixed following a previously described protocol [65]. Sixty specimens were selected at random from at least three representative microscopy fields and the number of units per specimen counted. A coccoid unit was considered double when at least an incipient constriction was recognizable. For autolysis experiments, cells were grown in THYE to OD_620_ = 0.5, chilled in an ice-water bath for 10 min, centrifuged at 3000 × g × 5 min at 4°C, resuspended in 1/10 of volume of cold THYE including 20% glycerol, and stored at −80°C until use. The cells were gently thawed on ice, resuspended as a 10-fold dilution in pre-warmed THYE, and incubated for 5 min at 37°C. After this time, 10 × MIC of vancomycin (2.5 μg ml^−1^) or penicillin (100 ng ml^−1^) were added and incubation allowed for 2 h at 37°C. Viable cell determinations were made on THYE plates containing 1% agar, incubated for 16 h at 37°C with 5% CO_2_. For LytA curation experiments, 10 × stock cells were resuspended in a 1:40 volume of pre-warmed THYE, pre-incubated for 5 min at 37°C, and then incubated for 30 min with 40 pM of purified LytA (a gift from Prof. Ernesto García) prior to the addition of 0.1% DOC.

### Animal model experiments

The effect of gene deletions on the establishment of pneumococcal sepsis was investigated using two groups of 5 CD-1 female mice (8–12 months old) as previously described [66]. Mixed infection experiments using a 1:1 ratio of the wild type and the isogenic mutant strain were used to determine the competitive index (CI), calculated as the number of mutant strain cells/wild type strain cells recovered from mice, divided by the number of mutant strain cells/wild type strain cells in the inoculum [67]. Every mouse was inoculated with a challenge suspension containing 2 × 10^4^ CFU of each strain. Bacteria were recovered from blood after 24 h of infection.

## Electronic supplementary material

Additional file 1: Table S1: Properties of selected proteins. (PDF 20 KB)

Additional file 2: Table S2: Putative nucleic-acid binding proteins. (PDF 85 KB)

Additional file 3: Table S4: Description of the domains in modular proteins listed in **Figure** 2 **.** (PDF 23 KB)

Additional file 7: **List of oligonucleotides used in this study.** (XLSX 34 KB)

Additional file 4: Table S3: List of HTEs published in the literature and considered in this study. (PDF 96 KB)

Additional file 5: Figure S1: HTE occurrence matrix for different assay types and conditions. (PDF 35 KB)

Additional file 6: **List of streptococcal strains considered in the calculation of the mean streptococcal identity of the proteins.** (PDF 22 KB)

## Abbreviations

cHP: Conserved hypothetical protein
CI: Competitive index
DOC: Deoxycholate
DUF: Domain of unknown function
HIC: Highly interacting and/or sequence conserved
HP: Hypothetical protein
HTE: High-throughput experiment
PPI: Protein-protein interaction.
