# Supplementary material for: Inspecting the potential physiological and biomedical value of 44 conserved uncharacterised proteins of Streptococcus pneumoniae
Source: BMC Genomics. 2014 Aug 5;15(1):652. doi: 10.1186/1471-2164-15-652 (PMC4143570; doi:10.1186/1471-2164-15-652)
Supplement: Supplementary file 1 — Additional file 1: Table S1: Properties of selected proteins. (PDF 20 KB) [file 12864_2013_6368_MOESM1_ESM.pdf]

Table S1 Properties of selected proteins.

R6 code[a]	Pfam domains	Operon	PPIs[b]	%I Strep[c]	Length (aa)
**Spr0004**	MMR_HSR1/DUF933	spr0001-12	24	94.1	371
**Spr0084**	Rhodanase/DUF3650	spr0084-5	1	83.7	328
Spr0175	DUF965	spr0175-7	9	89.0	88
Spr0177	DUF1292	spr0175-7	2	85.8	101
Spr0331	DUF1273	spr0331-6	2	62.4	175
Spr0391	DUF322	spr0390-2	6	68.8	129
Spr0399	DUF322	spr0399-400	10	84.7	121
**Spr0400**	Dak2/Dak1_2	spr0399-400	11	83.3	555
Spr0479	DUF448	spr0477-82	10	88.9	97
Spr0580	DUF436	spr0580-2	3	72.3	194
Spr0675	DUF1027	spr0675-8	12	58.1	176
Spr0705	ASCH	spr0700-5	3	72.7	146
**Spr0710**	Peptidase U32	None	2	77.1	356
Spr0747	Bmp	spr0743-7	8	71.5	374
**Spr0804**	DUF1980	spr0804-5	2	70.6	271
Spr0929	NA37	spr0922-32	3	73.4	335
Spr0930	Lysozyme-like	spr0922-32	2	64.4	204
**Spr0991**	HTH_24/DRTGG/CBS/4HBT	spr0989-92	4	65.4	425
Spr1000	DUF1831	spr0997-1003	3	73.8	115
Spr1010	YbaB_DNA_bd	None	3	82.0	99
Spr1035	DUF1694	None	0	47.9	148
Spr1158	DUF177	spr1157-8	2	57.4	180
Spr1268	DUF948	spr1267-70	4	61.5	127
Spr1327	DUF896	spr1327-9	2	90.4	85
**Spr1356**	TRP_16/TRP_7/TRP_12/Apc3/TRP_2	spr1356-7	1	64.3	414
Spr1418	YbbR/ YbbR	spr1415-20	3	50.8	259
Spr1423	UPF0052	spr1422-9	10	79.8	325
Spr1424	ATP_bind_2	spr1422-9	16	79.5	296
**Spr1506**	S4	spr1505-11	7	50.2	283
Spr1587	DUF795	spr1587-8	1	71.7	365
Spr1611	DUF3013	spr1609-11	2	67.4	156
**Spr1625**	DUF322	spr1623-6	4	72.2	202
Spr1658	DUF421	spr1658-9	1	80.4	176
Spr1719	DUF402	None	4	89.0	177
Spr1738	Transcriptional regulator	None	13	89.1	238
**Spr1782**	PDZ_2/Lon_C	spr1782-5	2	67.3	345
**Spr1798**	DUF258	spr1794-9	13	82.0	292
**Spr1806**	DUF1542/Gram_pos_anchor	None	0	66.5	221
Spr1810	Usp	None	0	70.8	171
**Spr1851**	KH_4/R3H	spr1850-3	6	62.4	328
Spr1865	DUF1033	spr1858-65	0	56.5	123
**Spr2010**	DHH/DHHA1	spr2007-10	4	63.8	657
**Spr2028**	HTH_25	spr2022-30	7	44.9	276
**Spr2030**	Peptidase_M16_C	spr2022-30	6	48.8	416

[a] Modular proteins are indicated in boldface.

[b] Protein-protein interactions (see Methods for details).
[c] Average identity shared by streptococci (see Methods for details).
