# Supplementary material for: Inspecting the potential physiological and biomedical value of 44 conserved uncharacterised proteins of Streptococcus pneumoniae
Source: BMC Genomics. 2014 Aug 5;15(1):652. doi: 10.1186/1471-2164-15-652 (PMC4143570; doi:10.1186/1471-2164-15-652)
Supplement: Supplementary file 2 — Additional file 2: Table S2: Putative nucleic-acid binding proteins. (PDF 85 KB) [file 12864_2013_6368_MOESM2_ESM.pdf]

Table S2 Putative nucleic acid binding proteins.

Code	*Nucleic acid molecule*	*Evidences of nucleic acid binding activity*[a]	*References*
Spr0004	RNA	Experimental/Structural (PDB code: 1JAL)	[1, 2]
Spr0479	RNA	Structural (PDB code: 1G2R)	[3]
Spr0675	DNA	8.3% BLAST-hits ("Transcriptional regulator")	
Spr0705	RNA	Structural (PDB code: 2DP9) / Pfam motif (ASCH; Pfam code: PF04266)	[4]
Spr0929	DNA	Experimental	[5]
Spr0991	DNA	Pfam motif (HTH-24; Pfam code: PF13412)	
Spr1010	DNA	Experimental/Structural (PDB code: 1YBX) / 95.3% BLAST-hits ("Nucleoid-associated protein")	[6, 7]
Spr1158	DNA	12.5% top-1000 BLAST-hits: "Nucleic-acid binding protein"	
Spr1327	DNA	Structural (PDB code: 2HEP)	[8]
Spr1506	RNA	Structural (PDB code: 3HUW) / Pfam motif (S4; Pfam code: PF01479)	[9]
Spr1738	DNA	Experimental/Structural/Pfam (YebC/PmpR; Pfam code: PF01709) / 17.4% BLAST-hits ("DNA-binding regulatory protein", "Probable transcriptional regulatory protein")	[10, 11]
Spr1851	RNA/ssDNA	Experimental/Structural (PDB code: 1MSZ)	[12]
Spr2028	DNA	Pfam motif (HTH-25; Pfam code: PF13413)	

[a] Representative structures were selected. The percentage of UNIPROT hits with descriptions compatible with nucleic-acid binding functions in the top-1000 BLAST is shown when above 5%.

References

1. Jain N, Dhimole N, Khan AR, De D, Tomar SK, Sajish M, Dutta D, Parrack P, Prakash B: ***E. coli* HflX interacts with 50S ribosomal subunits in presence of nucleotides**. *Biochem Biophys Res Commun* 2009, **379:**201-205.

2. Teplyakov A, Obmolova G, Chu SY, Toedt J, Eisenstein E, Howard AJ, Gilliland GL: **Crystal structure of the YchF protein reveals binding sites for GTP and nucleic acid**. *J Bacteriol* 2003, **185:**4031-4037.

3. Osipiuk J, Gornicki P, Maj L, Dementieva I, Laskowski R, Joachimiak A: ***Streptococcus pneumoniae* YlxR at 1.35 A shows a putative new fold**. *Acta Crystallogr D Biol Crystallogr* 2001, **57:**1747-1751.

4. Iyer LM, Burroughs AM, Aravind L: **The ASCH superfamily: novel domains with a fold related to the PUA domain and a potential role in RNA metabolism**. *Bioinformatics* 2006, **22:**257-263.

5. Murphy LD, Rosner JL, Zimmerman SB, Esposito D: **Identification of two new proteins in spermidine nucleoids isolated from *Escherichia coli***. *J Bacteriol* 1999, **181:**3842-3844.

6. Cooley AE, Riley SP, Kral K, Miller MC, DeMoll E, Fried MG, Stevenson B: **DNA-binding by *Haemophilus influenzae* and *Escherichia coli* YbaB, members of a widely-distributed bacterial protein family**. *BMC Microbiol* 2009, **9:**137.

7. Riley SP, Bykowski T, Cooley AE, Burns LH, Babb K, Brissette CA, Bowman A, Rotondi M, Miller MC, DeMoll E, Lim K, Fried MG, Stevenson B: ***Borrelia burgdorferi* EbfC defines a newly-identified, widespread family of bacterial DNA-binding proteins**. *Nucleic Acids Res* 2009, **37:**1973-1983.

8. Aramini JM, Sharma S, Huang YJ, Swapna GV, Ho CK, Shetty K, Cunningham K, Ma LC, Zhao L, Owens LA, Jiang M, Xiao R, Liu J, Baran MC, Acton TB, Rost B, Montelione GT: **Solution NMR structure of the SOS response protein YnzC from *Bacillus subtilis***. *Proteins* 2008, **72:**526-530.

9. Blaha G, Stanley RE, Steitz TA: **Formation of the first peptide bond: the structure of EF-P bound to the 70S ribosome**. *Science* 2009, **325:**966-970.

10. Liang H, Li L, Dong Z, Surette MG, Duan K: **The YebC family protein PA0964 negatively regulates the *Pseudomonas aeruginosa* quinolone signal system and pyocyanin production**. *J Bacteriol* 2008, **190:**6217-6227.

11. Shin DH, Yokota H, Kim R, Kim SH: **Crystal structure of conserved hypothetical protein Aq1575 from *Aquifex aeolicus***. *Proc Natl Acad Sci U S A* 2002, **99:**7980-7985.

12. Jaudzems K, Jia X, Yagi H, Zhulenkovs D, Graham B, Otting G, Liepinsh E: **Structural basis for 5'-end-specific recognition of single-stranded DNA by the R3H domain from human Smubp-2**. *J Mol Biol* 2012, **424:**42-53.
