# Supplementary material for: Inspecting the potential physiological and biomedical value of 44 conserved uncharacterised proteins of Streptococcus pneumoniae
Source: BMC Genomics. 2014 Aug 5;15(1):652. doi: 10.1186/1471-2164-15-652 (PMC4143570; doi:10.1186/1471-2164-15-652)
Supplement: Supplementary file 3 — Additional file 3: Table S4: Description of the domains in modular proteins listed in Figure 2 . (PDF 23 KB) [file 12864_2013_6368_MOESM3_ESM.pdf]

Table S4 Description for domains in modular proteins listed in Figure 2.

Protein	*Domain*	*Description*	*Begin*	*End*	*E-value*
Spr0004	MMR_HSR1	50S ribosome-binding GTPase	4	213	4.9e-21
Spr0004	YchF-GTPase_C	Protein of unknown function (DUF933)	287	370	3.2e-42
Spr0084	Rhodanese	Rhodanese-like domain	121	218	5.7e-12
Spr0084	DUF3650	Protein of unknown function DUF3650	291	318	1.7e-14
Spr0400	Dak2	DAK2 domain	34	199	5.0e-42
Spr0400	Dak1_2	Dihydroxyacetone kinase family	240	555	3.1e-126
Spr0710	Peptidase_U32	Peptidase family U32	121	354	1.2e-34
Spr0804	DUF1980	Domain of unknown function DUF1980	1	178	3.3e-65
Spr0991	HTH_24	Winged helix-turn-helix DNA-binding	1	50	1.8e-05
Spr0991	DRTGG	DRTGG domain	74	176	1.7e-31
Spr0991	CBS	CBS domain	185	241	2.8e-06
Spr0991	CBS	CBS domain	250	306	5.1e-07
Spr0991	4HBT	Thioesterase superfamily	342	416	9.4e-05
Spr1356	TPR_16	Tetratricopeptide repeat	41	102	9.4e-05
Spr1356	TPR_7	Tetratricopeptide repeat	137	168	8.3e-01
Spr1356	TPR_12	Tetratricopeptide repeat	167	237	1.5e-06
Spr1356	Apc3	Anaphase-promoting complex, cyclosome, subunit 3	283	363	6.4e-03
Spr1356	TPR_2	Tetratricopeptide repeat	379	404	4.6e-04
Spr1506	S4	S4 domain	205	251	4.9e-11
Spr1625	Asp23	Asp23 family	23	130	1.4e-29
Spr1782	PDZ_2	PDZ domain	105	196	2.3e-10
Spr1782	Lon_C	Lon protease (S16) C-terminal proteolytic domain	220	343	1.7e-06
Spr1798	DUF258	Protein of unknown function DUF258	123	274	1.5e-50
Spr1806	DUF1542	Domain of Unknown Function DUF1542	92	163	1.4e-09
Spr1806	Gram_pos_anchor	Gram positive anchor	179	217	3.0e-07
Spr1851	KH_4	KH domain	184	256	2.2e-20
Spr1851	R3H	R3H domain	273	326	4.5e-15
Spr2010	DHH	DHH family	333	496	7.7e-22
Spr2010	DHHA1	DHHA1 domain	585	644	8.4e-11
Spr2028	HTH_25	Helix-turn-helix domain	12	73	1.3e-20
Spr2030	Peptidase_M16_C	Peptidase M16 inactive domain	177	351	2.4e-23
