# Supplementary material for: Inspecting the potential physiological and biomedical value of 44 conserved uncharacterised proteins of Streptococcus pneumoniae
Source: BMC Genomics. 2014 Aug 5;15(1):652. doi: 10.1186/1471-2164-15-652 (PMC4143570; doi:10.1186/1471-2164-15-652)
Supplement: Supplementary file 5 — Additional file 4: Table S3: List of HTEs published in the literature and considered in this study. (PDF 96 KB) [file 12864_2013_6368_MOESM5_ESM.pdf]

Table S3 High-throughput experiments published in the literature considered in this work.

Experiment type	*Strain*	*Condition*	*Genes up*	*Genes down*	*Total*	*Reference*
ANTIGENome	TIGR4	ANTIGENome	97	0	97	[1]
Microarray	R6	Aerobiosis	54	15	69	[2]
Microarray	D39	Antimicrobial peptide bacitracin	81	66	147	[3]
Microarray	D39	Antimicrobial peptide LL-37	277	243	520	[3]
Microarray	D39	Antimicrobial peptide nisin	27	44	71	[3]
Microarray	R6	Chloramphenicol	53	103	156	[4]
Microarray	R6	CiaRH mutant	39	0	39	[5]
Microarray	R6	ClpP mutant	58	21	79	[6]
Microarray	D39	CodY	49	12	61	[7]
Microarray	TIGR4	CSP induction	124	64	188	[8]
Microarray	R6	Erytromycin	47	91	138	[4]
Microarray	D39	glnPA double mutant	74	12	86	[9]
Microarray	TIGR4	Incubation with host respiratory tract epithelial	56	47	103	[10]

		cells				
Microarray	R6	Low pH	50	76	126	[11]
Microarray	D39	LuxS mutant	47	0	47	[12]
Microarray	TIGR4	Manganese accumulation	87	85	172	[13]
Microarray	TIGR4	Manganese extracellular	13	39	52	[13]
Microarray	R6	Novobiocin (0.5xMIC)	364	364	728	[14]
Microarray	D39	Penicillin	81	131	212	[15]
Microarray	D39/TIGR4	PsaR	28	34	62	[16]
Microarray	R6	Puromycin	42	52	94	[4]
Microarray	R6	RitR mutant	37	16	53	[17]
Microarray	TIGR4	Serial Passage	67	26	93	[18]
Microarray	TIGR4	Temperature (21/29/33/40ºC)	629	0	629	[19]
Microarray	R6	Tetracyclin	41	87	128	[4]
Microarray	TIGR4	CSF infection	62	114	176	[20]
Microarray	TIGR4	Blood infection	83	52	135	[20]
Microarray	TIGR4	Epithelial cell contact	123	28	151	[20]

Microarray	T4	VncRS/vex mutants	45	34	79	[21]
STM	ST556 (19F)	Ear infection	131	0	131	[22]
STM	TIGR4	Lung Infection	387	0	387	[23]
STM	TIGR4	Meningitis	82	0	82	[24]
STM	ST556 (19F)	Nasal colonization	49	0	49	[22]

References

1. Giefing C, Meinke AL, Hanner M, Henics T, Bui MD, Gelbmann D, Lundberg U, Senn BM, Schunn M, Habel A, Henriques-Normark B, Ortqvist A, Kalin M, von GA, Nagy E: **Discovery of a novel class of highly conserved vaccine antigens using genomic scale antigenic fingerprinting of pneumococcus with human antibodies**. *J Exp Med* 2008, **205:**117-131.

2. Bortoni ME, Terra VS, Hinds J, Andrew PW, Yesilkaya H: **The pneumococcal response to oxidative stress includes a role for Rgg**. *Microbiology* 2009, **155:**4123-4134.

3. Majchrzykiewicz JA, Kuipers OP, Bijlsma JJ: **Generic and specific adaptive responses of *Streptococcus pneumoniae* to challenge with three distinct antimicrobial peptides, bacitracin, LL-37, and nisin**. *Antimicrob Agents Chemother* 2010, **54:**440-451.

4. Ng WL, Kazmierczak KM, Robertson GT, Gilmour R, Winkler ME: **Transcriptional regulation and signature patterns revealed by microarray analyses of *Streptococcus pneumoniae* R6 challenged with sublethal concentrations of translation inhibitors**. *J Bacteriol* 2003, **185:**359-370.

5. Mascher T, Zahner D, Merai M, Balmelle N, de Saizieu AB, Hakenbeck R: **The *Streptococcus pneumoniae* cia regulon: CiaR target sites and transcription profile analysis**. *J Bacteriol* 2003, **185:**60-70.

6. Robertson GT, Ng WL, Foley J, Gilmour R, Winkler ME: **Global transcriptional analysis of clpP mutations of type 2 *Streptococcus pneumoniae* and their effects on physiology and virulence**. *J Bacteriol* 2002, **184:**3508-3520.

7. Hendriksen WT, Bootsma HJ, Estevao S, Hoogenboezem T, de JA, de GR, Kuipers OP, Hermans PW: **CodY of *Streptococcus pneumoniae*: link between nutritional gene regulation and colonization**. *J Bacteriol* 2008, **190:**590-601.

8. Peterson SN, Sung CK, Cline R, Desai BV, Snesrud EC, Luo P, Walling J, Li H, Mintz M, Tsegaye G, Burr PC, Do Y, Ahn S, Gilbert J, Fleischmann RD, Morrison DA: **Identification of competence pheromone responsive genes in *Streptococcus pneumoniae* by use of DNA microarrays**. *Mol Microbiol* 2004, **51:**1051-1070.

9. Hendriksen WT, Kloosterman TG, Bootsma HJ, Estevao S, de GR, Kuipers OP, Hermans PW: **Site-specific contributions of glutamine-dependent regulator GlnR and GlnR-regulated genes to virulence of *Streptococcus pneumoniae***. *Infect Immun* 2008, **76:**1230-1238.

10. Song XM, Connor W, Hokamp K, Babiuk LA, Potter AA: ***Streptococcus pneumoniae* early response genes to human lung epithelial cells**. *BMC Res Notes* 2008, **1:**64.

11. Martin-Galiano AJ, Overweg K, Ferrandiz MJ, Reuter M, Wells JM, de la Campa AG: **Transcriptional analysis of the acid tolerance response in *Streptococcus pneumoniae***. *Microbiology* 2005, **151:**3935-3946.

12. Joyce EA, Kawale A, Censini S, Kim CC, Covacci A, Falkow S: **LuxS is required for persistent pneumococcal carriage and expression of virulence and biosynthesis genes**. *Infect Immun* 2004, **72:**2964-2975.

13. Rosch JW, Gao G, Ridout G, Wang YD, Tuomanen EI: **Role of the manganese efflux system *mntE* for signalling and pathogenesis in *Streptococcus pneumoniae***. *Mol Microbiol* 2009, **72:**12-25.

14. Ferrandiz MJ, Martin-Galiano AJ, Schvartzman JB, de la Campa AG: **The genome of *Streptococcus pneumoniae* is organized in topology-reacting gene clusters**. *Nucleic Acids Res* 2010, **38:**3570-3581.

15. Rogers PD, Liu TT, Barker KS, Hilliard GM, English BK, Thornton J, Swiatlo E, McDaniel LS: **Gene expression profiling of the response of *Streptococcus pneumoniae* to penicillin**. *J Antimicrob Chemother* 2007, **59:**616-626.

16. Hendriksen WT, Bootsma HJ, van DA, Estevao S, Kuipers OP, de GR, Hermans PW: **Strain-specific impact of PsaR of *Streptococcus pneumoniae* on global gene expression and virulence**. *Microbiology* 2009, **155:**1569-1579.

17. Ulijasz AT, Andes DR, Glasner JD, Weisblum B: **Regulation of iron transport in *Streptococcus pneumoniae* by RitR, an orphan response regulator**. *J Bacteriol* 2004, **186:**8123-8136.

18. Pandya U, Sinha M, Luxon BA, Watson DA, Niesel DW: **Global transcription profiling and virulence potential of *Streptococcus pneumoniae* after serial passage**. *Gene* 2009, **443:**22-31.

19. Pandya U, Allen CA, Watson DA, Niesel DW: **Global profiling of *Streptococcus pneumoniae* gene expression at different growth temperatures**. *Gene* 2005, **360:**45-54.

20. Orihuela CJ, Radin JN, Sublett JE, Gao G, Kaushal D, Tuomanen EI: **Microarray analysis of pneumococcal gene expression during invasive disease**. *Infect Immun* 2004, **72:**5582-5596.

21. Haas W, Sublett J, Kaushal D, Tuomanen EI: **Revising the role of the pneumococcal *vex-vncRS* locus in vancomycin tolerance**. *J Bacteriol* 2004, **186:**8463-8471.

22. Chen H, Ma Y, Yang J, O'Brien CJ, Lee SL, Mazurkiewicz JE, Haataja S, Yan JH, Gao GF, Zhang JR: **Genetic requirement for pneumococcal ear infection**. *PLoS One* 2008, **3:**e2950.

23. Hava DL, Camilli A: **Large-scale identification of serotype 4 *Streptococcus pneumoniae* virulence factors**. *Mol Microbiol* 2002, **45:**1389-1406.

24. Molzen TE, Burghout P, Bootsma HJ, Brandt CT, van der Gaast-de Jongh CE, Eleveld MJ, Verbeek MM, Frimodt-Moller N, Ostergaard C, Hermans PW: **Genome-wide identification of *Streptococcus pneumoniae* genes essential for bacterial replication during experimental meningitis**. *Infect Immun* 2011, **79:**288-297.
