# Supplementary material for: Inspecting the potential physiological and biomedical value of 44 conserved uncharacterised proteins of Streptococcus pneumoniae
Source: BMC Genomics. 2014 Aug 5;15(1):652. doi: 10.1186/1471-2164-15-652 (PMC4143570; doi:10.1186/1471-2164-15-652)
Supplement: Supplementary file 6 — Additional file 5: Figure S1: HTE occurrence matrix for different assay types and conditions. (PDF 35 KB) [file 12864_2013_6368_MOESM6_ESM.pdf]

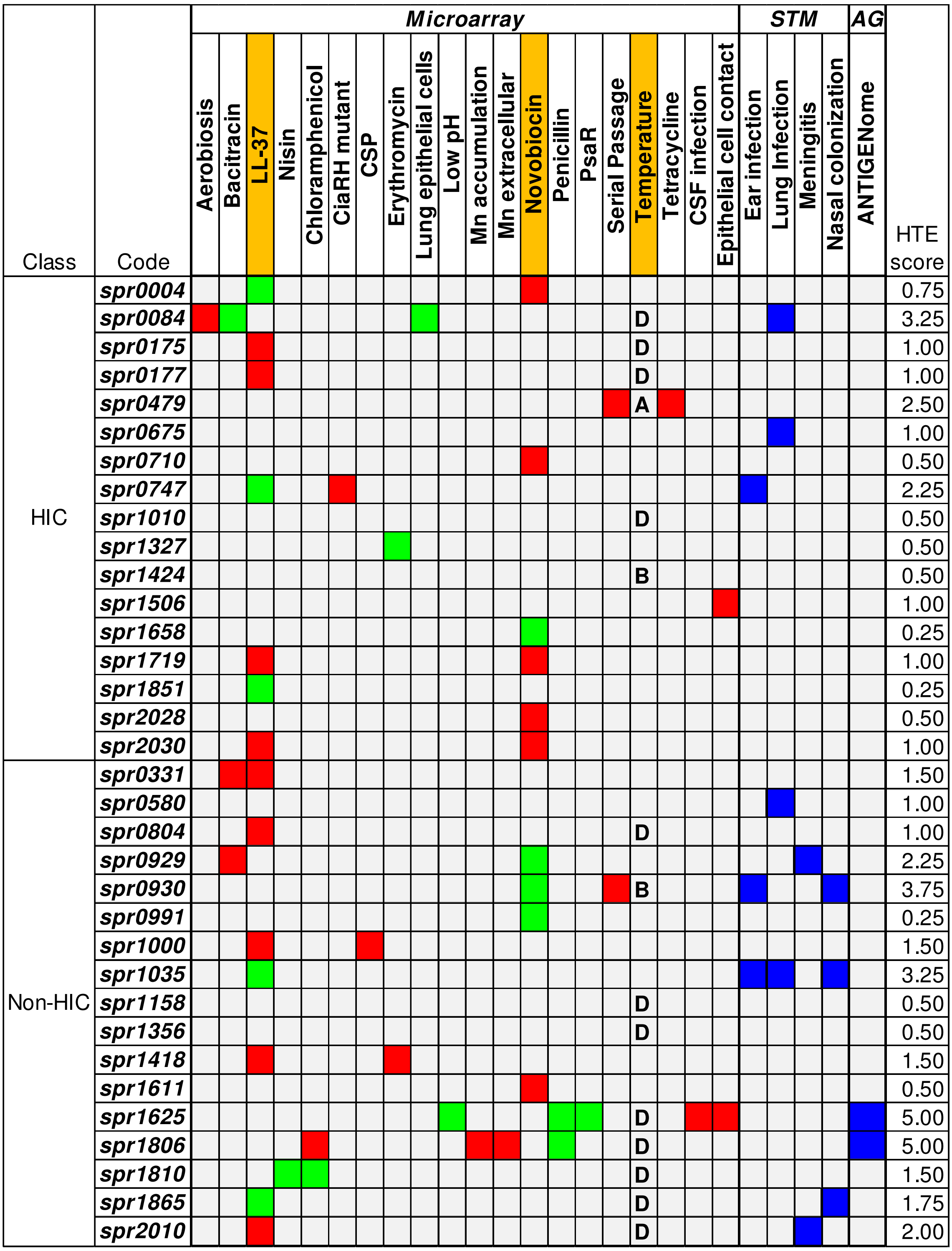

Figure S1 HTE occurrence matrix under different assay types and conditions. Up-(red cells) and down-(green cells) regulation in microarray experiments. Presence (blue cells) in STM or antigenome assays. Response to temperature: A, B and D genes enhance expression at 33ºC, 40ºC and 21ºC, respectively [1]. Conditions in orange involve more than 500 genes, a fact that was considered to calculate the HTE-score. Only conditions affecting members of the protein list of this work and genes with HTE scores>0 are shown. For further details see Methods and Additional file 3: Table S3.

References

1. Pandya U, Allen CA, Watson DA, Niesel DW: **Global profiling of *Streptococcus pneumoniae* gene expression at different growth temperatures.** *Gene* 2005, **360**: 45-54.
