# Supplementary material for: Inspecting the potential physiological and biomedical value of 44 conserved uncharacterised proteins of Streptococcus pneumoniae
Source: BMC Genomics. 2014 Aug 5;15(1):652. doi: 10.1186/1471-2164-15-652 (PMC4143570; doi:10.1186/1471-2164-15-652)
Supplement: Supplementary file 7 — Additional file 6: List of streptococcal strains considered in the calculation of the mean streptococcal identity of the proteins. (PDF 22 KB) [file 12864_2013_6368_MOESM7_ESM.pdf]

Streptococcus agalactiae 2603
Streptococcus agalactiae A909
Streptococcus agalactiae NEM316
Streptococcus dysgalactiae subsp. *equisimilis* GGS_124
Streptococcus equi subsp. 4047
Streptococcus equi subsp. *zooepidemicus*
Streptococcus equi subsp. *zooepidemicus* MGCS10565
Streptococcus gallolyticus UCN34 uid46061
Streptococcus gordonii Challis substr *CH1*
Streptococcus mitis B6
Streptococcus mutans NN2025
Streptococcus mutans UA159
Streptococcus pyogenes M1 GAS
Streptococcus pyogenes MGAS10270
Streptococcus pyogenes MGAS10394
Streptococcus pyogenes MGAS10750
Streptococcus pyogenes MGAS2096
Streptococcus pyogenes MGAS315
Streptococcus pyogenes MGAS5005
Streptococcus pyogenes MGAS6180
Streptococcus pyogenes MGAS8232
Streptococcus pyogenes MGAS9429
Streptococcus pyogenes NZ131
Streptococcus pyogenes SSI-1
Streptococcus pyogenes Manfredo
Streptococcus sanguinis SK36
Streptococcus suis 05ZYH33
Streptococcus suis 98HAH33
Streptococcus suis BM407
Streptococcus suis P1_7
Streptococcus suis SC84
Streptococcus thermophilus CNRZ1066
Streptococcus thermophilus LMD-9
Streptococcus thermophilus LMG 18311
Streptococcus uberis 0140J

List of streptococcal strains with complete genomic sequences considered to calculate the average streptococcal identity.
